# Factors limiting immunization coverage in urban Dili, Timor-Leste

**DOI:** 10.9745/GHSP-D-13-00115

**Published:** 2013-11-14

**Authors:** Ruhul Amin, Telma Joana Corte Real De Oliveira, Mateus Da Cunha, Tanya Wells Brown, Michael Favin, Kelli Cappelier

**Affiliations:** a MCHIP-Maternal and Child Health Integrated Program, John Snow, Inc., Washington, DC, USA; b The Ministry of Health, Dili, Timor-Leste; c U.S. Agency for International Development, Dili, Timor-Leste

## Abstract

**Background:**

Timor-Leste's immunization coverage is among the poorest in Asia. The 2009/2010 Demographic and Health Survey found that complete vaccination coverage in urban areas, at 47.7%, was lower than in rural areas, at 54.1%. The city of Dili, the capital of Timor-Leste, had even lower coverage (43.4%) than the national urban average.

**Objective:**

To better understand the service- and user-related factors that account for low vaccination coverage in urban Dili, despite high literacy rates and relatively good access to immunization services and communication media.

**Methods:**

A mixed-methods (mainly qualitative) study, conducted in 5 urban sub-districts of Dili, involved in-depth interviews with18 Ministry of Health staff and 6 community leaders, 83 observations of immunization encounters, 37 exit interviews with infants' caregivers at 11 vaccination sites, and 11 focus group discussions with 70 caregivers of vaccination-eligible children ages 6 to 23 months.

**Results:**

The main reasons for low vaccination rates in urban Dili included caregivers' knowledge, attitudes, and perceptions as well as barriers at immunization service sites. Other important factors were access to services and information, particularly in the city periphery, health workers' attitudes and practices, caregivers' fears of side effects, conflicting priorities, large family size, lack of support from husbands and paternal grandmothers, and seasonal migration.

**Conclusion:**

Good access to health facilities or health services does not necessarily translate into uptake of immunization services. The reasons are complex and multifaceted but in general relate to the health services' insufficient understanding of and attention to their clients' needs. Almost all families in Dili would be motivated to have their children immunized if services were convenient, reliable, friendly, and informative.

## Abstract (precis)

Simple access to immunization services does not necessarily translate into uptake of services. In Timor-Leste, key determinants of the success of vaccination efforts are health workers' attitudes, the manner in which patients are treated, aspects of service organization, adequate supply of vaccines, and caregivers' basic knowledge about immunization.

## BACKGROUND

The Democratic Republic of Timor-Leste is one of the world's newest nations. A former Portuguese colony, Timor-Leste was occupied by Indonesia in 1975 and restored to independence on May 20, 2002.

Timor-Leste's health and development indicators, including immunization coverage, are among the poorest in Asia. The 2009/2010 Demographic and Health Survey found immunization coverage for Timor-Leste to be 66.7% for DTP3 (third dose of diphtheria, pertussis, and tetanus vaccine) and 68.2% for measles. In 7 of the total 13 districts, BCG (bacillus Calmette–Guérin) coverage was less than 85%. Nationally, 22.7% of 1-year olds in Timor-Leste had never received any vaccination. Complete vaccination coverage was lower in the urban areas (47.7%) than in rural areas (54.1%). Dili, the capital city of Timor-Leste, had an even lower rate of complete vaccination coverage, at 43.4%, than the average urban coverage.^1^

Timor-Leste is one of the world's newest nations. Its health and development indicators, including immunization coverage, are among the poorest in Asia.

Dili district has 6 sub-districts and 31 villages/*sucos* (administrative sub-divisions). Atauro, the only rural sub-district, is an island approximately 30 kilometers off the coast of Dili town.^2^ Dili has come to have the largest urban concentration in Timor-Leste due to rapid in-migration since independence.^3^ The report of the 2010 Timor-Leste Population Census noted that 21.9 % of the country's population lives in the district of Dili, most of them in urban areas.^2^ The Timor-Leste Survey of Living Standards (2007) found that the urban population of the country has better housing, easier access to hospitals and clinics, schools and public transportation, and higher education levels.^4^

Timor-Leste's Ministry of Health (MOH) operates at 4 levels—central, district, sub-district, and community. Services are provided at a national hospital in Dili, 5 referral hospitals, 67 community health centers (CHCs) (1 in each sub-district), and 192 health posts (HPs) in different *sucos*.^5^ In 2008 the SISCa (*Servisu Integradu da Saúde Communitária*), monthly integrated outreach sessions, were added to the system structure to provide every *suco* with access to integrated health services, including immunization.^6^ Today, most immunizations are given at CHCs and during monthly SISCa sessions.

Besides the National Hospital in Dili, there are 5 CHCs, 9 HPs, 20 SISCa, and several private clinics.^7^ The national hospital provides only birth doses of BCG and polio vaccines. The MOH has estimated that private clinics (for-profit and nonprofit) deliver one-fourth of basic health services, but few of them offer immunization services.^5^

Although the Expanded Programme on Immunization (EPI) has made significant progress in Timor-Leste since the country emerged from decades of turmoil in 1999, issues with immunization coverage and quality persist. Since 2008, the MOH has tried to improve the quality of immunization services through enhanced pre-service and refresher training and supportive supervision.^8^ Despite these initiatives, several factors that hindered immunization coverage were recognized, including minimal community participation, vaccinators' lack of interpersonal communication skills, and deficient routine data recording and reporting to serve as a solid basis for District Health Services (DHS) to increase coverage.

As everywhere, a multitude of factors influence health care-seeking behavior in Timor-Leste. These include deeply rooted cultural beliefs and practices, levels of education and health knowledge, service accessibility, gender roles, and out-of-pocket expenses for clients. Although government health services are free, there are out-of-pocket expenses associated with transportation and loss of earnings. Also, most women in Timor-Leste depend on their husbands' income, and, therefore, the husband is the decision-maker.^9^ Average walking time from households to the nearest health facility is about 70 minutes,^10^ but the walk is much longer for some families. Particularly during the wet season, access to services in rural areas may be blocked by overflowing rivers and poor road conditions.

Because of its large population (234,026 in 2010),^2^ Dili district contains more unvaccinated and partially vaccinated children than any other district in the country.^11^ Since epidemics often start—or spread rapidly—in densely populated areas (as was the case with Timor-Leste's measles outbreak in 2011), it is important for children throughout Timor-Leste to raise coverage in Dili. Yet, as mentioned, despite good physical access to immunization services, vaccination coverage rates in urban areas are puzzlingly, often unaccountably, lower than rates in rural areas.^12^

Vaccination coverage rates in urban areas are puzzlingly, often unaccountably, lower than rates in rural areas.

The objective of this study was to identify the key factors that contribute to low immunization coverage in urban Dili. The findings were intended to help the Dili DHS and partners to devise effective and feasible solutions that would improve immunization services, reduce dropout rates, and increase coverage. The study sought to:

1. Determine deficiencies/insufficiencies within the health services that contribute to sub-optimal vaccination coverage
2. Better understand parents' knowledge, attitudes, and practices regarding vaccinations and the health system and how these may contribute to sub-optimal vaccination coverage
3. Recommend modifications to service availability, provider practices, community mobilization, and/or health promotion that could improve vaccination coverage

## METHODS

### Study Design

A cross-sectional, mixed-methodology study conducted in March and April 2012 combined qualitative (primarily) and quantitative methods, including observations, exit interviews, in-depth interviews, and focus group discussions.

### Study Population and Sampling

A total of 83 immunization encounters were observed, and 37 exit interviews were conducted with caregivers. Observations and exit interviews took place at 11 sites (5 CHCs, 3 SISCa, 1 HP, 1 private clinic, and the national hospital). These sites included all CHCs in urban Dili, the only national hospital, and the largest private clinic that immunizes. The 3 SISCa were selected randomly, one each from high, medium, and low immunization coverage sub-areas. Researchers observed either up to 20 children vaccinated or for 60 minutes, whichever came first. Caregivers were selected for exit interviews randomly at each site after seen by health care providers.

We conducted 24 in-depth interviews with health staff members (11 vaccinators and 7 health facility directors) and community leaders (6 *suco* chiefs). Health staff members were randomly chosen from all 5 CHCs, the national hospital, and 1 private clinic. Community leaders also were selected randomly from each group of *sucos* with poor, average, and good immunization coverage.

Family members (mothers, fathers, and grandmothers) of children ages 6 to 23 months participated in focus group discussions. To determine eligibility by children's immunization status and type of caregivers, we screened these participants using a structured questionnaire and classified them into 3 groups:

- **No immunization:** Child had no immunizations at all.
- **Fully immunized:** Child had all of the immunizations that he/she was eligible for at his/her age.
- **Partially immunized:** Child had some, but not all, of the immunizations that he/she was eligible for at his/her age.

The 26 urban *sucos* were segmented by immunization coverage levels, and 11 *sucos* were randomly selected from these 26 for screening and selection of focus group discussion participants. These 11 *sucos* included 2 from the high-coverage category, 4 from the medium category, and 5 from the low-coverage category.

Participants were selected at random from a starting point in each selected neighborhood; each researcher went in opposite directions and screened every third household. The interviewer explained the study and asked eligible participants to provide verbal consent to participate voluntarily. In total, 70 randomly selected households were identified.

Table 1 reports details on the study sample. The only rural sub-district of Dili, Atauro, was excluded from the study population.

**Table 1.** Study Sample

**Methodology and Types of Participants**	**No. of Participants**
Observed Immunization Encounters	
Mothers	69
Fathers	3
Mothers and fathers together	4
Other caregivers	7
**Subtotal**	**83**
Exit Interviews	
Caregivers	37
**Subtotal**	**37**
In-Depth Interviews	
Health staff	18
Community leaders	6
**Subtotal**	**24**
Focus Group Discussions	
Mothers	52
Fathers	10
Grandmothers	8
**Subtotal**	**70**
**TOTAL**	**214**

### Observations

Using structured checklists, experienced and trained teams observed vaccination sessions. The observations focused on characteristics of caregivers, types of antigens offered, potential missed opportunities, and health workers' manner, counseling, and vaccination technique.

### Exit Interviews

To learn the caregivers' perspectives on communication and their interactions with health care workers, the researchers conducted up to 5 exit interviews with caregivers selected randomly as they were leaving each observed vaccination site. These interviews also allowed the interviewers to understand how well the caregivers remembered the information given to them.

The team used a semi-structured questionnaire that focused on waiting time, level of client satisfaction, what immunizations the child received, providers' communication and behavior toward clients, reasons for bringing the child, the return date for the next vaccination, and understanding of possible adverse events following immunization.

### In-Depth Interviews With Health Staff

We used a semi-structured questionnaire to facilitate in-depth interviewers with health staff (vaccinators and health facility directors). Topics included, but were not limited to, perceptions, level of knowledge, suggestions on how immunization services can be improved, reasons that some children are not vaccinated, seasonal migration, role of the vaccinator in informing the community about services, understanding of the community's role in vaccination services, and MOH and DHS support.

### In-Depth Interviews With Community Leaders

Community leaders were interviewed using a semi-structured questionnaire to understand their perspective and their role in vaccination activities. We collected data on community demographics, leaders' role in the community and the health of the community, community challenges, relationship with government health services/systems, interaction with private health services, perceptions of childhood immunization, knowledge of immunization services in the community, and the role of community leaders in immunization services.

### Focus Group Discussions

Focus groups ranged in size from 2 to 9 people, and the discussions lasted from 1 to 1.5 hours. We collected information on perceptions of immunizations, experiences with immunization services, reasons for current immunization status, and suggestions for how immunization services can be improved.

### Data Analysis

For quantitative analysis, we entered data from observations and exit interviews into Microsoft Excel and conducted a simple descriptive frequency analysis.

**Figure 1.**
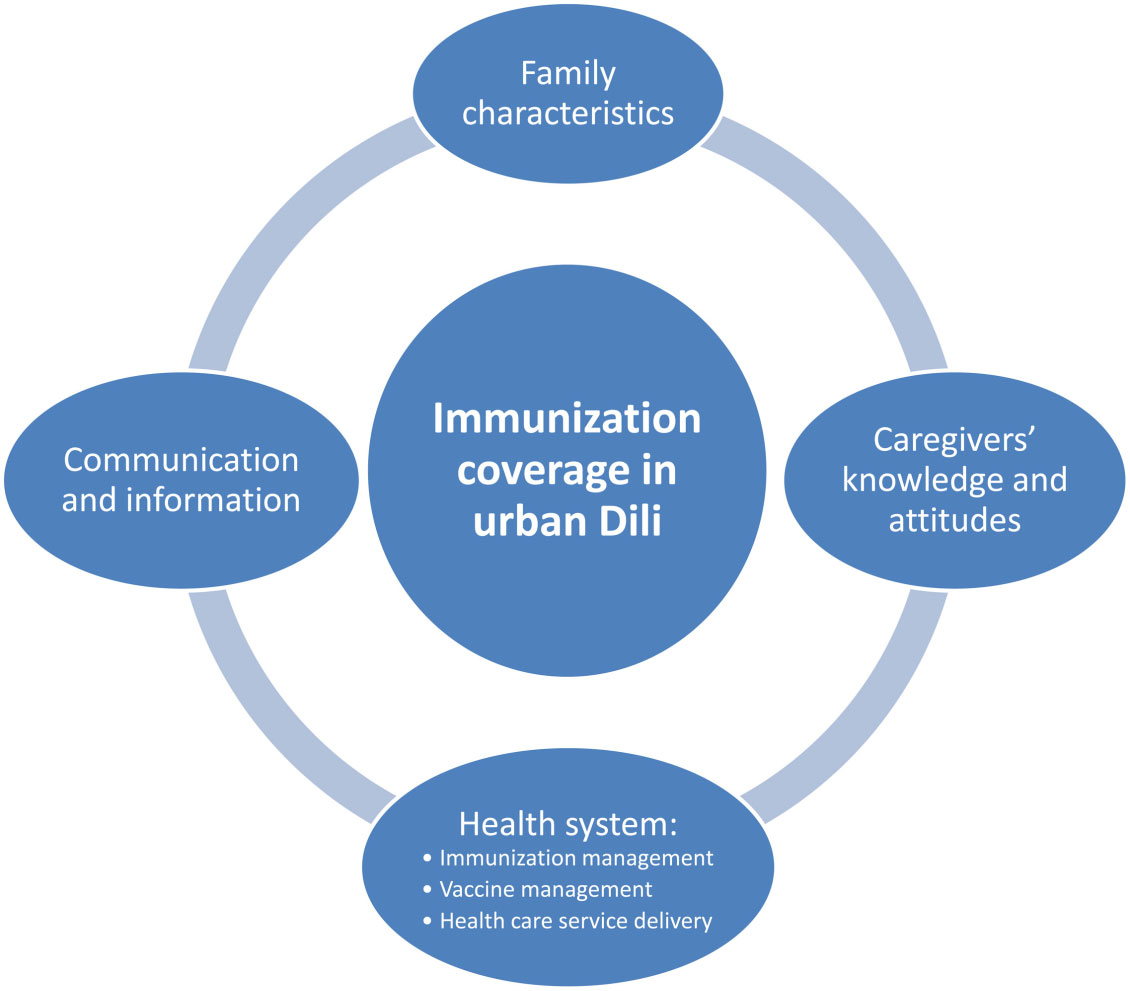
Four Themes Associated With Immunization Coverage in Urban Dili, Timor-Leste

Qualitative information collected through exit interviews, in-depth interviews, and focus group discussions was transcribed, translated into English, and analyzed using a manual coding system. The data analysis process followed a sequence of interrelated steps, such as reading, coding, displaying, summarization, and interpretation. After cross-checking for validity and credibility through daily meetings and discussions, the team identified 4 common themes: family characteristics, caregivers' knowledge and attitudes, the health system, and communication and information (Figure 1).^13^

### Ethical Consideration

We obtained ethical clearance from the Essex Institutional Review Board, USA, and the Research and Development Cabinet of the MOH, Timor-Leste. Before data collection, we obtained verbal consent from the respondents.

## RESULTS

Below, we present the results of focus group discussions, observations, exit interviews, and in-depth interviews.

### Family and Socioeconomic Characteristics

**Figure 2.**
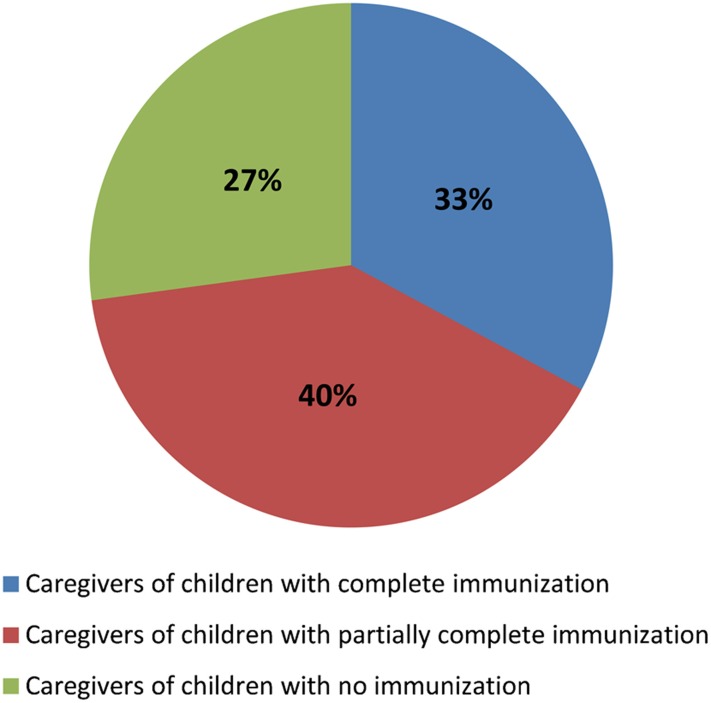
Immunization Status of Caregivers' Children Among caregivers who participated in focus group discussions (N = 70).

Among the caregivers (N = 70) who participated in focus groups, 33% had children/grandchildren with complete immunization, whereas 40% had partially immunized children/grandchildren, and 27% had children/grandchildren with no immunization (Figure 2).

Of the 52 mothers who participated in the focus group discussions, 50 were housewives, with families of up to 12 children. One mother studied at the university, and 1 worked as a public servant. Most of mothers had never attended school or had limited education (up to primary school). Most (n = 46) had very temporary work in farming, small business, construction, and/or other manual labor.

Caregivers from more densely populated areas of Dili were found to have better access to information and communication from various sources, such as health facilities, neighbors, SISCa, media, and community leaders.

Many mothers, regardless of their socioeconomic status, remarked that they were willing to pay up to US$3.00 for transportation or US$30.00 for consultations in private clinics in order to get their children vaccinated or treated for illness. Caregivers said that 5 private clinics in Dili requested payment for vaccination, while government clinics provided free vaccination.

Caregivers are often too busy to take their children or grandchildren for immunization. For both employed and unemployed mothers, cultural gatherings, seasonal migration, and employment or domestic duties appear to have a higher priority than obtaining preventive health services. Many families move back to their home villages during the rainy season for agriculture purposes.

**Figure 3.**
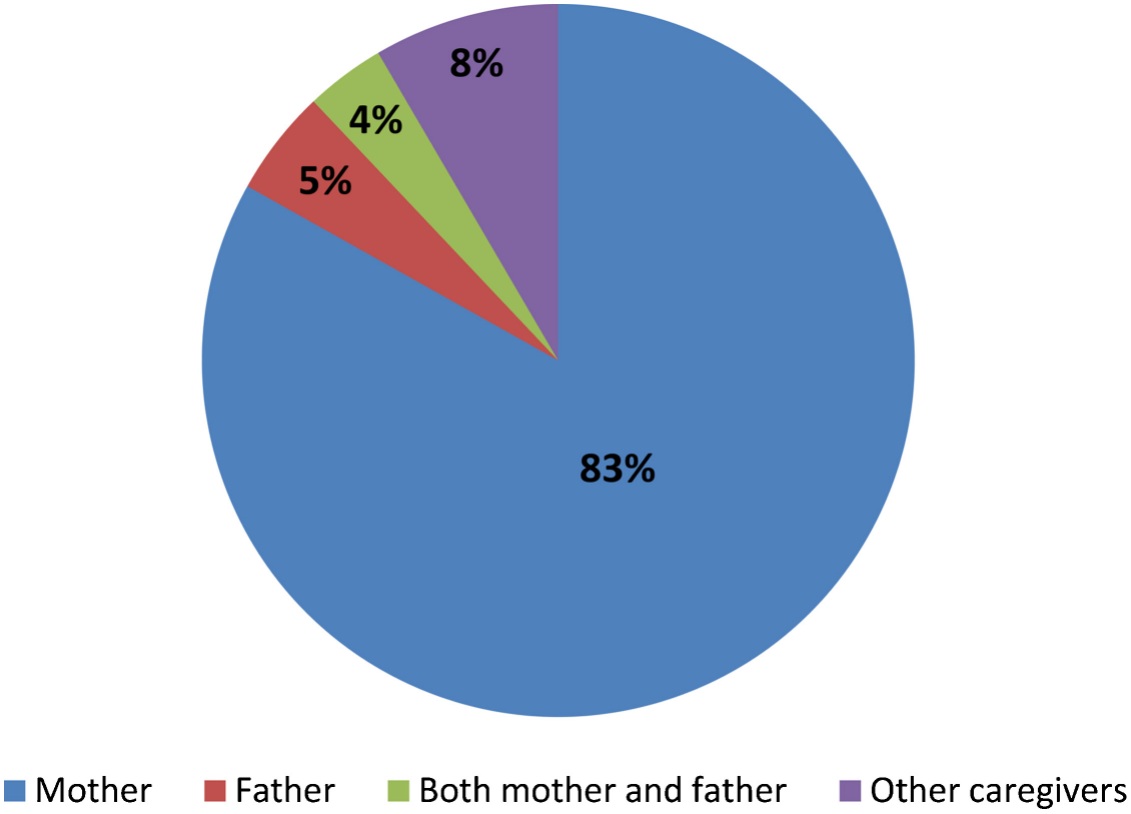
Relationship of Caregiver to Child Taken for Immunization Based on analysis of health facility observations (N = 83).

Analysis of health facility observations show that mothers (83%) were the most likely household member to take their children for immunization (Figure 3).

### Caregivers' Knowledge and Attitudes

During the discussions, caregivers of fully immunized children were able to cite the benefits of immunization, although few could explain how vaccination works, and few were familiar with the vaccination schedule. Mothers of children who were fully immunized received more support (financial and moral) from their husbands and family members, and they were more likely to prioritize their children's health needs than mothers of children who were not immunized or partially immunized. Paternal grandmothers were very supportive of children's immunization and were often involved in the decision about when and where to seek services for immunization. Fathers were very unlikely to object to children being immunized.

Caregivers with partial and unimmunized children often did not complete their children's vaccinations because of negative experiences with health care services.

Caregivers with partial and unimmunized children often did not complete their children's vaccinations because of negative experiences with health care services. One caregiver reported that one health care worker told her:

“It's better not to bring your child here (health facility). … Sometimes you [the caregiver] come regularly and sometimes you don't. … So it's better not to bring your child back again. …” We felt very bad.

Those who had a bad experience with one child would not take other children for vaccination. A few mothers explained that they were shouted at when they came late or if they had lost their LISIO book (*Livrinho Saude Inan no Oan,* or Mother and Child Health Book). Some mothers were afraid of taking their children if they had missed an appointment and would rather avoid going back than face interrogation.

Others were discouraged to return for subsequent vaccinations after their children suffered from adverse events following immunization (such as fever, crying, or insomnia) or wasted a visit because the vaccine was not available. One father said in a focus group discussion:

I wanted to take my child. … My second and third child received immunization here. … And then my children got very high fever all day and night. … I was the one who was afraid.

Other reasons that children were only partially immunized included caregivers not realizing that they needed to bring their children back for additional immunizations, child illnesses, and many mothers having job responsibilities.

Women who had delivered at home without a skilled birth attendant said they were scared of being shouted at by the health worker for birthing at home, so they did not seek treatment or vaccination for their children afterwards. Women who recalled having a negative experience during childbirth at a health facility were less likely to return to a health clinic for postnatal checkups or for vaccination.

Some caregivers of unimmunized children mentioned that they were reluctant to have their children vaccinated or that they lived too far from services to have their children vaccinated. A small number of caregivers thought that immunizations were harmful for their children, and they did not believe that vaccination could prevent diseases. Again, complications after previous vaccinations also contributed to low interest among these caregivers in having their children immunized. Table 2 summarizes the reasons for a child being fully, partially, or unimmunized.

**Table 2.** Reasons for Child Having Complete, Partial, and No Immunizations, Compiled From Focus Group Discussions

**Sociocultural Factors**	**Fully Immunized**	**Partially Immunized**	**Not Immunized**
Understand the benefits	✓	✓	
Motivated	✓		
Collaboration with husband	✓		
Conflicting priorities (working parents)		✓	
Afraid, shy		✓	
Misunderstood schedule and came late		✓	
Children got ill		✓	
Raining and distance		✓	
Bad experiences^a^		✓	✓
Perception that child is too weak for vaccination			✓
False beliefs that vaccination does not prevent diseases			✓
Lost health card or no card		✓	
Lack of interest or motivation			✓
Delivered at home			✓

a Includes fear of provider or of interrogation, adverse events, unavailable vaccine, and miscellaneous reasons.

### Health Workers' Views, Attitudes, and Practices

**Figure 4.**
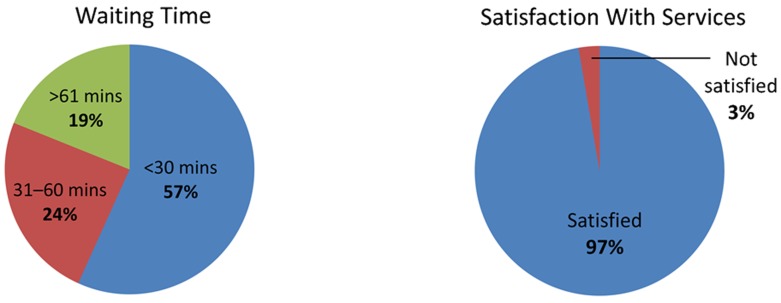
Caregivers' Assessments of Waiting Times and Satisfaction With Services Based on analysis of data from exit interviews (N = 37).

During observations, health care workers appeared to be friendly and respectful to mothers and their children. Nearly all mothers (97%) during exit interviews said that they were satisfied with the services received, even though 43% had waited more than 30 minutes (Figure 4).

**Figure 5.**
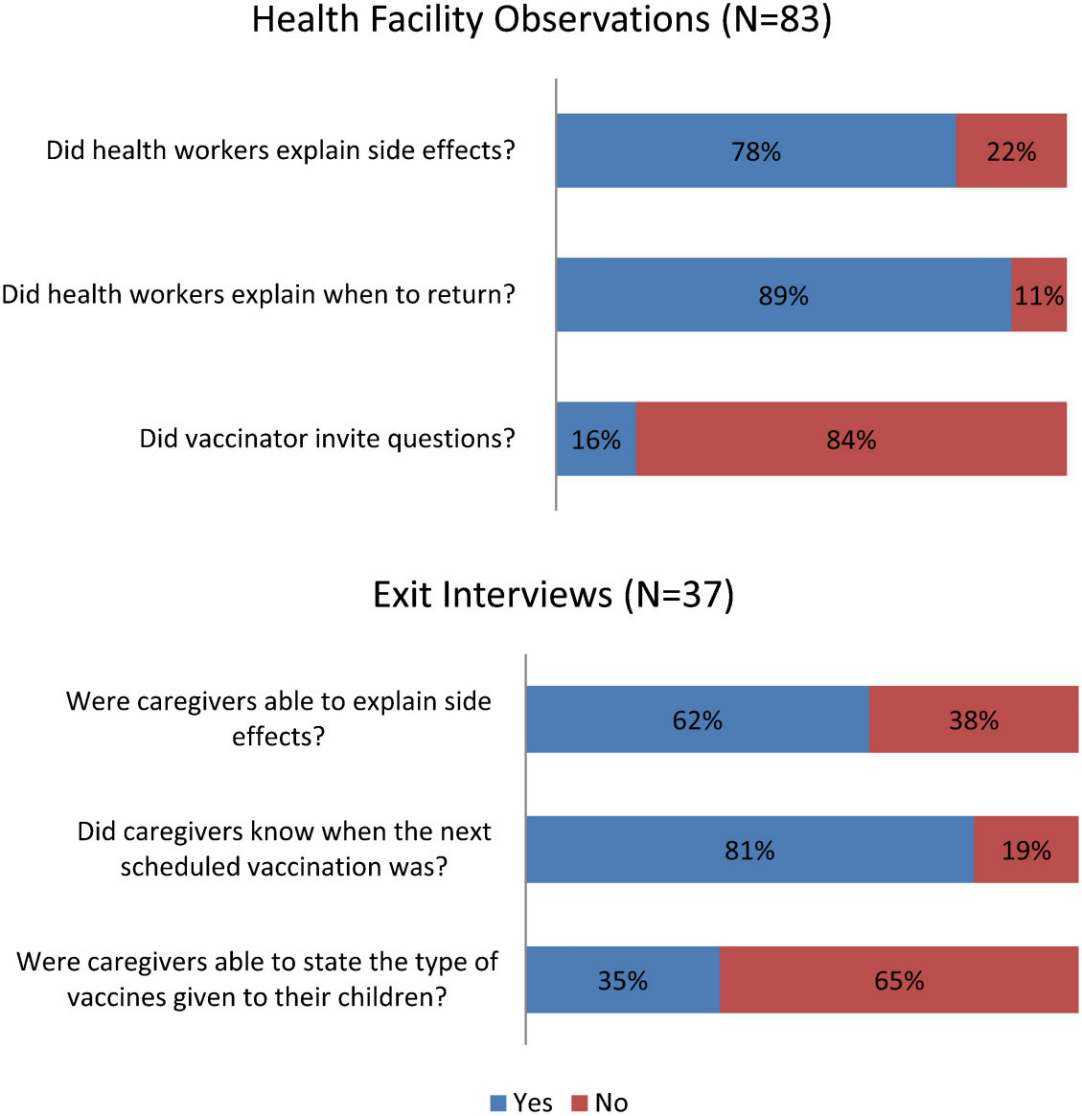
Quality of Counseling and Health Education

Vaccinators' counseling of clients was observed to be of reasonable quality, although it fell short of what health staff members were taught in training: 78% of clients received information on side effects; 89% were advised on when to return, but only 16% were invited to ask questions. These observations were consistent with responses from caregivers during exit interviews, in which most clients (62%) were able to explain the side effects (fever, swelling at the injection site, diarrhea); 81% could give the date for next immunization (for example, in 1 month); but 65% were unable to state the type and benefits of the vaccine administered to their children (Figure 5). Therefore, it seems, although health care workers provided some counseling for caregivers, the communication and information provided was frequently incomplete.

The quality of counseling was reasonable, although counseling fell short of what health staff members had been taught.

A caregiver said in a focus group discussion:

In reality and based on my experiences when I took my children for immunization … When they [health workers] finished vaccination, they have never explained what type of vaccine was given to my child and what was the benefit of vaccination. Was it vaccine-preventable diseases? They did not explain. They only vaccinated my child and just told me to come back next month. … That's it.

This was highlighted in the discussions when caregivers said that they often misunderstood the schedule for future vaccinations, and as a result some children failed to complete the schedule.

Most health workers have multiple tasks in the clinic. Of the 18 health care workers interviewed, 9 were aware of immunization coverage, and 3 were aware of dropout rates for their health facilities. Half of them reported that they were not only providing immunization services at health facilities but also were involved in house-to-house screening and vaccination activities, outreach programs, and care for pregnant women. All 18 declared that their multi-task functions, in addition to the shortage of health workers, limited their ability to deliver better quality immunization services. Nevertheless, 13 said that they always provided counseling to the caregivers.

### Service Provision

Health care workers said that there are not enough workers and transport for outreach activities, and they are not regularly updated as needed to provide an adequate standard of care.

Interviews with health staff and facility directors found that many health facilities, particularly in harder-to-reach areas of Dili, lack a consistent schedule of vaccination sessions and of regular outreach sessions. Some health facilities offered only certain antigens on certain days of the week and/or limited the number of caregivers who could obtain any health care service during each morning or afternoon. Most of the participating health facilities would attend to a maximum of 50 patients in the morning and then reopen for vaccination and other health services in the afternoon. In spite of health clinics being open from 8 am to 5 pm, most patients are seen in the morning. Many health facilities offer BCG and measles vaccines only a few days per week to avoid vaccine wastage; for example, vaccinators do not want to open a 20-dose BCG vial for only a few children. In observed sessions, 2 children who were ill but eligible to be vaccinated were refused vaccinations.

Health care workers claimed in the in-depth interviews that, when waiting children could not be vaccinated, they always encourage frustrated parents to bring their children back. Parents could be particularly frustrated if they had missed work, traveled long distances, spent money for transportation, and waited for a long time only to find that their child could not be vaccinated that day.

At observation sites, the study found that waiting time and venue (small, crowded, and dirty) were not issues for most caregivers as long as their children received the immunization.

Many discussion participants talked about difficult access to health services for families in areas further from facilities and with no outreach. Unexpectedly, the team found that outreach programs conducted by the MOH, such as SISCa, had never reached some families in Dili district. Even in Dili, geography and walking distance, especially during the rainy season, are barriers to bringing children to be vaccinated. Respondents, both community leaders and caregivers, expressed their wishes to have health programs and immunization more accessible to their community.

Outreach programs had never reached some families in Dili.

### Health Information and Education

Most respondents in less densely populated areas of Dili (the city periphery and areas where people live on mountainsides) reported that they did not know where and when to obtain immunization information or services. This report is consistent with community leaders' views, which emphasized that some caregivers had inadequate information and communication about immunization services. Caregivers, in particular, think that information currently available is not sufficient.

This lack of practical information, added to a lack of accessibility, acceptability, and affordability of services in some parts of Dili, affects people's perceptions of the barriers and benefits of immunization and eventually discourages them from seeking vaccination. The main sources of information on immunization and support reported by the respondents were their peers, their own experiences, mass media, and print materials (such as pamphlets and posters).

## DISCUSSION

Studies on the reasons for low immunization coverage from a variety of countries have identified such factors as inadequate immunization services, poor parental knowledge and attitudes, limited access to services, poor health staff attitudes and practices, unreliability of services, false contraindications, fears of side effects, conflicting priorities, and parental beliefs.^14^^–^^17^

Similarly, this study indicates that poor immunization coverage in Dili is related to multiple, complex, and interrelated factors, including inconsistent and irregular immunization sessions, lack of adequate outreach activities, and some health care workers' poor behavior toward clients, which leads mothers to fear being reprimanded. Underlying these factors is the health system's problems in providing adequate resources to facilities to conduct the full range of services, including integrated outreach services. User factors also contribute to low immunization coverage, including primary caregivers being busy with other obligations and families' incomplete understanding of the benefits of vaccination.

The study found that caregivers' negative experiences at vaccination sites or with post-immunization side effects were among the most common factors that discouraged immunization. Such findings are commonly reported elsewhere.^18^ While some research finds that caregivers who have a negative experience with health care workers are less likely to follow the vaccination schedule, this is not always the case.^18^ In Dili, health care workers' attitudes and behavior toward clients appear to have a large influence over whether clients return.

Health care workers' attitudes and behavior toward clients appear to influence clients' decisions to return for vaccination.

Despite the national service standard that all vaccinations should be available at CHCs every day,^19^ this study encountered limitations on the availability of immunization services. Facilities restricted certain antigens to certain days and limited the number of persons attended in a session. Frequent stockouts, too, appeared to lead to missed opportunities for vaccination and incomplete and delayed vaccination. Another study in Timor-Leste indicates that this situation occurs not only in Dili but also in other districts and is a major reason for limiting immunization coverage in the country.^20^ Immunizations (all antigens) should be offered every day at all CHCs, as the MOH Basic Package of Health Services specifies.

Service availability and access are likely to be worse in the city periphery or less densely populated sub-areas or mountainous areas, where communities are sparse. Although perceptions of distance among urban caregivers in Dili are not clear, this issue appears to be related to immunization status. This finding is also seen in other studies. For example, a study in Bangladesh found that women who reported having a health facility nearby (<1 km) were more likely to fully immunize their children.^21^ Another study, in India, found a positive association between the presence of a health center within 2 km of an urban slum and the immunization status of children.^22^ Further studies are needed to understand the perception of caregivers of urban Dili about distance to immunization and other health services.

The lack of regular outreach activities or SISCa in urban Dili limited the uptake of services. Many mothers in Dili are working, at least in short-term jobs. Extended clinic hours for immunization would likely help these working mothers. Studies have found that extended hours can reduce dropouts and left-outs in urban areas.^16^^,^^23^ In addition, making services more reliable, for example, by having regular stocks of vaccines, is crucial to ensuring the community's faith in service delivery.

Caregivers of fully immunized children had good basic knowledge and understanding of immunization. Studies show that knowledge gaps underlie low compliance with vaccination schedules.^24^

Seasonal migration to and from urban Dili is quite common and affects immunization coverage. Rural-urban migration—for example, where families move for better economic opportunities—has been shown to adversely affect use of health services, including immunization.^25^ As people move from one community to another, they lose track of the time for vaccination, children are left with other caregivers, or parents forget the immunization records. This problem warrants further study in Timor-Leste. Tracking and reporting systems could be established for children who receive vaccines from sites other than their designated sites. These systems could trace these children for the subsequent vaccinations.

Paternal grandmothers in Dili were very supportive of immunization and were often involved in the decision about when and where to seek service for immunization. As in many other countries in South Asia,^15^ mothers may play a subsidiary role to the paternal grandparents in decision-making on seeking immunization services for children. Mothers need both financial and moral support from their husbands to avail their children of immunization services.

Paternal grandmothers—key decision-makers in families—were supportive of immunization.

### Limitations

We used a variety of qualitative methods to obtain an in-depth understanding of the determinants of under-immunization and to enable triangulation of findings from different informants and situations (for example, mothers in exit interviews and mothers in focus group discussions; heath care workers and mothers). Data from observation and exit interviews enable some frequency analysis quantitatively, but it does not permit statistical testing.

Discussion group participants were from poor and middle-income families. Thus, the beliefs and attitudes of rich families are missing from the study findings. The research team's presence probably encouraged the health care workers being observed to be friendly and respectful to mothers and children, a bias known as the Hawthorne effect.^26^ Information from focus group discussions, where many mothers claimed to have been humiliated by vaccinators, painted quite a different picture.

### Recommendation for the MOH and the Dili DHS

This study supports the recommendation that EPI service hours should be extended.^27^ Moreover, Dili needs more outreach sessions. These could be organized at schools, through night clinics, and after church on Sundays or at other times. In order to maximize service delivery and optimize use of limited resources, these outreach efforts could integrate other maternal and child services as well. Health facilities should, according to MOH standards, provide immunization services every day that the facilities are open. The MOH needs to ensure an uninterrupted supply of vaccines and associated supplies around the year. Currently, health services do little to promote vaccination or to engage with community leaders and networks.

EPI service hours should be extended. Moreover, Dili needs more outreach sessions.

To improve coverage, the district health services and the MOH would benefit from taking steps to improve health care workers' attitudes and practices toward clients and to expand mobilization activities. These improvements may require a combination of training, including sensitization aimed at changing attitudes, supportive supervision, steps to reduce the flood of clients at certain times of the day, and adding additional staff. Improved health care worker communication can help caregivers understand what vaccinations their child has received and should receive in the future, and can reduce anxiety about side effects. Health care workers should focus on explaining to parents that some side effects are normal, that simple treatment methods are available, and that these side effects mean that the vaccination is working.

EPI microplanning was just beginning in Dili at the time of this study. Microplanning at the sub-district level should be organized regularly and include community leaders, health care workers, volunteers, and civil society organizations. The national and district immunization programs need to support Dili's sub-districts in communicating better about vaccination—their importance, safety, and the basic schedule.

## CONCLUSIONS

Good access to health facilities or health services does not necessarily translate to uptake of services, and this is as true for immunization as for any other preventive service. This study found that in Dili district, health care workers' attitudes, the way that health care workers behave with clients, and convenient (client-centered) provision of immunization services are extremely important to maintaining caregivers' motivation to fully immunize their children. We also found that a basic understanding of immunization, such as its general purpose and the need for several visits, is a key factor in the completion of all vaccinations for infants. In addition to these generally expected factors affecting uptake of immunization services, we also discovered some unexpected findings, including the lack of outreach and health education in Dili, grandmothers' role in decision-making, caregivers' perceptions and beliefs, and seasonal migration. The reasons that children are not fully vaccinated are complex and multifaceted, and so the solutions must be, also.
